# Differential alpha-gal expression during *Amblyomma hebraeum* and *Rhipicephalus evertsi* tick feeding and development: A driver for the development of alpha-gal syndrome in South Africa

**DOI:** 10.1016/j.jacig.2025.100623

**Published:** 2025-12-06

**Authors:** Tatenda Murangi, Ben Mans, Ronel Pienaar, Maresa Botha, Heidi Facey-Thomas, Stephen Cunningham, Ali Halajian, Lokesh Joshi, Franco H. Falcone, William Horsnell, Michael E. Levin

**Affiliations:** a Division of Immunology and Wellcome Centre for Infectious Diseases Research in Africa (CIDRI), Institute of Infectious Disease and Molecular Medicine, University of Cape Town, Cape Town, South Africa; b Epidemiology, Parasites and Vectors, Agricultural Research Council–Onderstepoort Veterinary Research, Pretoria, South Africa; c Department of Life and Consumer Sciences, University of South Africa, Florida, South Africa; d Division of Paediatric Allergology, University of Cape Town, Cape Town, South Africa; e Advanced Glycoscience Research Cluster, University of Galway, Galway, Ireland; f Research Administration and Development, University of Limpopo, Sovenga, South Africa; g Institute of Parasitology, Biomedical Research Center Seltersberg (BFS), Justus Liebig University Giessen, Giessen, Germany; h Medical Research Council Centre for Medical Mycology, University of Exeter, Exeter, United Kingdom

**Keywords:** Alpha-gal, food allergy, galactose-α-1,3-galactose, red meat allergy, ticks, *Amblyomma hebraeum*, *Rhipicephalus evertsi*, IgE

## Abstract

**Background:**

The presence of galactose-α-1,3-galactose (alpha-gal) in tick salivary antigens has been implicated to initiate host IgE responses resulting in sensitization to alpha-gal.

**Objective:**

We sought to investigate the presence of alpha-gal in different anatomic locations in native South African tick species at different feeding and developmental stages and the ability of sera from our allergic cohort to bind to these tick proteins.

**Methods:**

Alpha-gal–containing proteins in laboratory-reared ticks at different feeding and developmental stages were detected through Western blotting and immunohistochemical staining. IgE and IgG4 toward the tick proteins were analyzed via ELISA.

**Results:**

There is differential expression of alpha-gal in endemic South African ticks *Amblyomma hebraeum* and *Rhipicephalus evertsi*. Immunoblotting demonstrated an increase in the prominence of alpha-gal–containing protein bands in salivary glands to be proportional to feeding time. Alpha-gal in both fed and unfed ticks was localized in the salivary acini and testes. IgE and IgG4 to *A hebraeum* antigens were significantly raised in alpha-gal–allergic individuals. There was a correlation between anti–*A hebraeum* IgE and anti–alpha-gal IgE in the alpha-gal–allergic group. Inhibition of human serum anti–alpha-gal IgE to *A hebraeum* and *R evertsi* proteins was significantly reduced by the addition of bovine thyroglobulin.

**Conclusions:**

We anticipate repeated exposure to differentially expressed *A hebraeum* and *R evertsi* alpha-gal–containing salivary proteins during feeding to cause alpha-gal sensitization.

Alpha-gal syndrome (AGS) is an IgE-mediated hypersensitivity reaction to the carbohydrate galactose-α-1,3-galactose (alpha-gal), a major constituent of glycoproteins and glycolipids in nonprimate mammals. Allergic reactions to alpha-gal are characterized by either immediate reactions to parenterally administered drugs^1^ or a delayed hypersensitivity reaction after oral consumption of food or use of pharmaceutical products that contain alpha-gal.^2^^,^^3^ An association between AGS and specific tick species has been established,^4^, ^5^, ^6^, ^7^, ^8^, ^9^ with initial findings arising from an overlap between patients developing type I hypersensitivity reactions to a colorectal cancer drug, cetuximab, and those presenting with delayed hypersensitivity reactions to red meat after bites with the tick *Amblyomma americanum*.^10^

The induction of anti–alpha-gal IgE in patients with AGS after tick bites but not after dermal exposure to alpha-gal–containing proteins alone (eg, by butchers/meat handlers) implies that exposure alone may not be sufficient and there may be adjuvant properties of tick saliva capable of promoting IgE production against harmless substances as well as disruption of regulatory pathways. The morbidity associated with AGS has been linked to the direct effect of IgE responses to tick salivary antigens containing the alpha-gal epitope.^3^^,^^4^^,^^6^^,^^11^ Studies show evidence of alpha-gal epitopes in salivary glands of partially fed and fully fed *A americanum* and *Ixodes scapularis*,^5^ *I ricinus*,^12^ *A sculptum*,^13^ and *Haemaphysalis longicornis*.^6^ Alpha-gal epitopes have also been shown in the midguts of ticks belonging to the family *Ixodes*, particularly *I ricinus* and *I scapularis.* Although once fed, alpha-gal epitopes became undetectable.^5^^,^^8^

However, not all tick species have been reported to have alpha-gal. Crispell et al^5^ showed the absence of binding of anti–alpha-gal antibodies with unfed and partially fed salivary glands from *A maculatum* and *Dermacentor variabilis* as well as saliva antigens from the former.^5^ Although numerous tick species have been identified as clinically relevant to the development of AGS in populations across North and South America, Europe, and Asia, no specific tick has yet been definitively linked to the onset of clinical AGS on the African continent. We have previously reported the presence of alpha-gal in 2 South African ticks, *A hebraeum* and *Rhipicephalus evertsi*, collected in the vicinity of our alpha-gal–allergic cohort.^14^ To further understand at which point these native tick species may cause alpha-gal sensitization, we investigated the presence of alpha-gal in South African native ticks at different feeding and developmental stages as well as examined the binding of tick proteins with sera from alpha-gal–allergic individuals in Mqanduli, Eastern Cape province, South Africa.

## Methods

### Participants

Subjects with alpha-gal allergy proven by oral food challenge were identified in Mqanduli district, Eastern Cape province, South Africa, as previously described.^15^ Age-matched controls from the same area were identified. Briefly, 72 participants with a history of symptoms after the consumption of red meat were enrolled along with 21 participants who had no history of adverse reactions to red meat and were regularly consuming meat. Blood samples were collected from all participants to test for total IgE and specific IgE antibodies (by using ImmunoCAP testing; Phadia) to alpha-gal antigen with results expressed in kilounits per liter. Sera were analyzed using the ImmunoCAP100 (Thermo Fisher Scientific, Uppsala, Sweden), with a lower detectable level of 0.35 kU/L. Alpha-gal–positive participants were described as having a positive ImmunoCAP result (≥0.35 kU/L) and symptoms after an oral food challenge to cooked beef sausage. Alpha-gal–negative participants were described as individuals with a negative ImmunoCAP result (<0.35 kU/L) and no symptoms and/or individuals with a positive ImmunoCAP result and no reaction after an oral food challenge. The study was approved by the Human Research Ethics Committee of the University of Cape Town (174/2017) and informed consent, parental consent, and assent were obtained from all participants. Serum samples from participants were kept at −80°C until further use.

### *A hebraeum* and *R evertsi* acquisition and preparation

To investigate the presence of alpha-gal at different feeding stages, the Agricultural Research Council–Onderstepoort Veterinary Research laboratory-reared *R evertsi* and *A hebraeum* colonies were expanded as previously described.^16^ For this study, female and male ticks were grouped into 3 categories: unfed, partially fed (partially engorged), and fully fed (engorged) on the basis of the degree of blood meal intake. These were fed on the back of South African meat merino sheep weighing 92 kg at a ratio of 1.7 ticks/kg bodyweight with defined protocols in an academic veterinary center.^16^ Detached fully engorged ticks were removed with a spoon from the inside of the calico bags attached to the sheep’s back. To collect partially fed ticks, fine curved forceps were used to grab the ticks’ mouthparts and detach them from the host. After collection, ticks were separated from excreta and other debris by using a fine sieve. Running tap water was used to wash away blood or other animal fluid exuded from feeding wounds. The ticks were left to dry thoroughly on filter paper. Adult ticks at different feeding stages were dissected to collect salivary glands and guts.

Protein extraction was done in TRIS-HCl (pH 8.0; Sigma-Aldrich, St Louis, Mo) by sonication and pelleting of cell membranes and debris by centrifugation at 16,000*g*, before bicinchoninic acid assay protein quantification and storage of the protein lysates at −80°C until needed. Whole ticks at different feeding and developmental stages were also frozen down at −80°C until further use.

### Detection of alpha-gal in *A hebraeum* and *R evertsi*

Immunoblotting was performed using 5 μg of protein from each antigen preparation. The samples were separated on a 10% SDS-PAGE gel at 120 to 150 V and transferred to a 0.4-μm nitrocellulose membrane (Bio-Rad) at 80 V for 2 hours. Ponceau S (Sigma-Aldrich) staining was carried out to validate wet transfer. The nitrocellulose membrane was blocked with 3% BSA Fraction V (molecular biology grade, ≥98% purity; Carl Roth, Karlsruhe, Germany) in PBS (Thermo Fisher Scientific) at room temperature on the laboratory bench, followed by overnight incubation with alpha-gal specific antibody, namely, anti–alpha-gal chicken scFv antibody (1:5000).^17^ Detection of binding to alpha-gal was achieved by the addition of Biotin anti–6-His tag as a secondary antibody (1:5000) and streptavidin–horseradish peroxidase conjugate (1:5000) with 3 to 5 washes for 5 minutes of the blot with TRIS-buffered saline-Tween 20 (Sigma-Aldrich) in between antibody blot incubations. Pork kidney protein homogenates were used as positive controls for alpha-gal.

Immunohistochemistry was carried out on *A hebraeum* and *R evertsi* sections preserved in 4% formaldehyde (Sigma-Aldrich) before embedding in paraffin wax blocks. Cut sections of 4 μm were hydrated in varying concentrations of ethanol and blocked with 3% H_2_O_2_ (Sigma-Aldrich) for 15 minutes. Citrate buffer (0.1 M; pH 6.0; Sigma Aldrich) was used for antigen retrieval for 2 minutes in a pressure cooker. Tick sections were blocked with 1% BSA Fraction V (Carl Roth) for optimal staining conditions. Staining for alpha-gal was done by adding an anti–alpha-gal chicken scFv antibody (1:200) on whole cut sections in an overnight incubation at 4°C. Detection of binding was performed by sequentially adding Biotin anti–6-His tag (BioLegend, San Diego, Calif) as a secondary antibody (1:1000), and streptavidin–horseradish peroxidase (1:400) (BioLegend). VIP substrate (Vector Laboratories, Newark, Calif) was used for color development, producing a purple reaction product in positive samples. Slides were counterstained with methylene green, dehydrated, and cover-slipped.

### Human sera IgE and IgG4 binding with *A hebraeum and R evertsi* antigen

IgG4 and IgE antibodies against *A hebraeum* antigens were measured using an indirect ELISA, and 96-well Nunc Maxisorp plates (Thermo Fisher Scientific) were coated overnight at 4°C with 20 μg/mL of *A hebraeum* antigen diluted in 50 mM carbonate buffer at pH 9.6. Plates were washed 3 times with 200 μL PBS/0.05% Tween-20. After blocking with 200 μL of 5% BSA Fraction V (Carl Roth) for 2 hours at 37°C, plates were washed another 3 times and incubated with sera from participants in triplicates at a dilution of 1:10 in PBS for another 90 minutes at 37°C. After another 3 washes, bound antibodies were incubated with biotinylated mouse anti–human IgG4/IgE antibodies (Southern Biotech) at a dilution of 1:1000 in PBS–Tween-20 in 5% BSA at 4°C overnight. Plates were washed 3 times, followed by 1 final wash with PBS without Tween-20. Ultra 3,3',5,5'-Tetramethylbenzidine-ELISA (Thermo Fisher Scientific) was used for visualization (incubated for 30 minutes at 37°C). Reactions were stopped by addition of 50 μL 2 M sulfuric acid and absorbance measured at 450 nm in a spectrophotomer (CLARIOstar Plus; BMG Labtech, Ortenberg, Germany).

Inhibition of anti–alpha-gal IgE binding to *A hebraeum* (adult and larval whole tick lysate, unfed salivary glands, and unfed guts) and *R evertsi* (adult and larval whole-body lysate) antigens by 2 mg/mL bovine thyroglobulin (BTG; Merck, Darmstadt, Germany) was assessed by ELISA as previously described.^14^ The ELISA setup was as described earlier; however, to inhibit anti–alpha-gal IgE binding, the plates were incubated with 50 μL 1:10 sera diluted in PBS in the presence or absence of 2 mg/mL BTG.

### Statistical analysis

Analysis of antibody levels to *A hebraeum* and *R evertsi* between participants with and without alpha-gal allergy was done using GraphPad Prism v8 (GraphPad Software, La Jolla, Calif). Mann-Whitney *U* tests were used to test the difference in the IgE and IgG4 between the allergic groups. Correlation analysis was done using the Pearson correlation coefficient (*r*). To evaluate the difference in sera binding to tick proteins before and after BTG inhibition, a Wilcoxon test was carried out. Groups were judged to be significantly different if the *P* value was less than .05 (∗*P* ≤ .05; ∗∗*P* ≤ .01; ∗∗∗*P* ≤ .001; ∗∗∗∗*P* ≤ .0001).

## Results

### Alpha-gal is differentially expressed during feeding and development of *A hebraeum* and *R evertsi*

**Fig 1:**
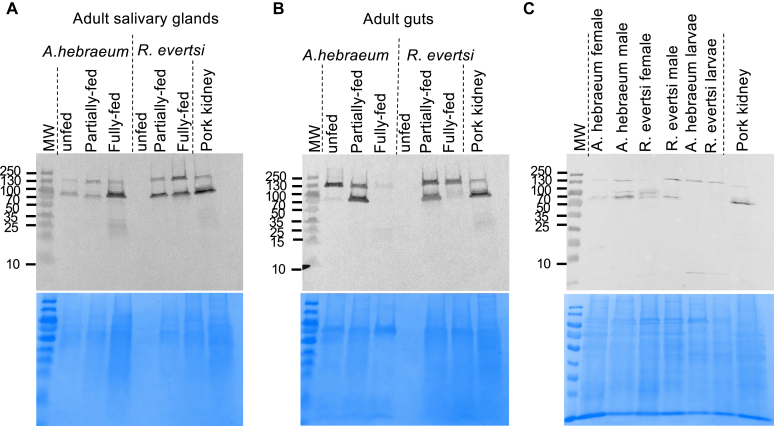
**A-C,** Differential expression of alpha-gal proteins in *A hebraeum* and *R evertsi*. Immunoblotting of 5 μg salivary gland (Fig 1, *A*) and gut (Fig 1, *B*) at different feeding stages and unfed whole tick proteins from the adult and larval developmental stages probed with an anti–alpha-gal scFv chicken antibody (Fig 1, *C*). Pork kidney was used as a positive control. Data shown are representative of 3 independent experiments.

Alpha-gal–containing proteins in salivary glands from adult *A hebraeum* were recognized at 2 bands—one at 130 kDa and the other at 70 kDa (Fig 1, *A*). However, despite these bands being present in all the feeding stages, the prominence of the bands increased proportionally to feeding time. Salivary glands from adult *R evertsi* showed the same trend.

Guts from adult *A hebraeum* showed a single band in the unfed state at 130 kDa, 2 bands at 130 and 70 kDa in the partially fed state, and no bands in the fully fed state (Fig 1, *B*). In *R evertsi*, alpha-gal–containing proteins in the partially fed state produced bands at 130 and 70 kDa, whereas only 1 protein band at 130 kDa was detected for guts in the fully fed state (Fig 1, *B*). Pork kidney showed bands at 130 and 70 kDa.

Next, we investigated the effect of *A hebraeum* and *R evertsi* developmental stage on the presence of alpha-gal in these homogenates. Homogenates from larval ticks (both *A hebraeum* and *R evertsi*) showed alpha-gal–containing proteins at 130 kDa only, which corresponds to one of the bands seen in adult *A hebraeum* and *R evertsi* homogenates (Fig 1, *C*).

### Immunolocalization of alpha-gal in *A hebraeum* and *R evertsi* at different feeding stages

**Fig 2:**
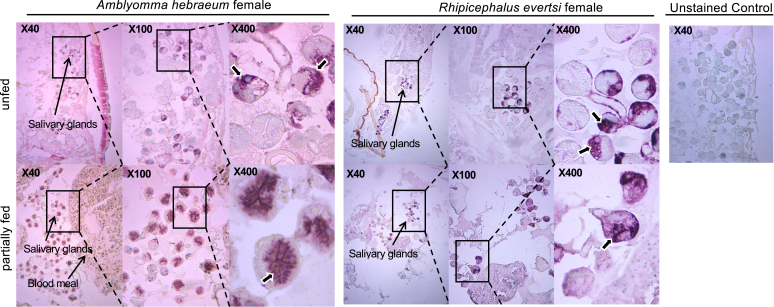
Immunolocalization of alpha-gal in adult female *A hebraeum and R evertsi* at different feeding stages. Sections (4 μm) of female *A hebraeum* and *R evertsi* ticks at unfed and partially fed feeding stages were stained for alpha-gal using anti–alpha-gal chicken scFv antibody. These were imaged at 40×, 100×, and 400× magnification. Data are representative of 2 experiments with 2 section replicates per slide. A *thick black arrow* indicates positive staining.

**Fig 3:**
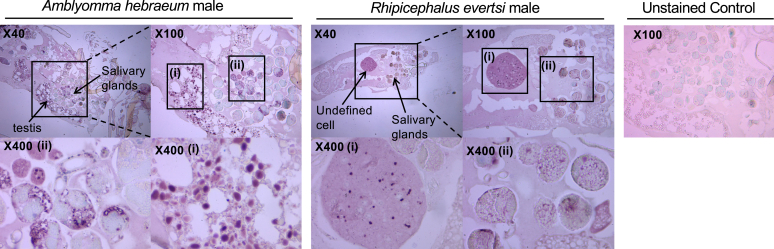
Immunolocalization of alpha-gal in unfed male adult *A hebraeum and R evertsi* ticks. Sections (4 μm) of *A hebraeum* and *R evertsi* male ticks were stained for alpha-gal using anti–alpha-gal chicken scFv antibody. These were imaged at 40×, 100×, and 400× magnification. Data are representative of 2 experiments with 2 section replicates per slide. An *arrow* indicates positive staining.

To investigate the effect of feeding on the localization of alpha-gal, we made sections from unfed and partially fed ticks. Unfed and partially fed female ticks (Fig 2) showed staining for alpha-gal in the salivary acini. In partially fed *A hebraeum* female ticks, a red-brownish mass was present, but it was not positive for alpha-gal staining (Fig 2). Unfed *A hebraeum* male ticks showed alpha-gal staining in their salivary acini and testes (Fig 3), whereas unfed *R evertsi* male ticks showed alpha-gal staining in the undefined cells and salivary glands (Fig 3).

### Anti–*A hebraeum* IgE and IgG4 are raised in alpha-gal–allergic patients

**Fig 4:**
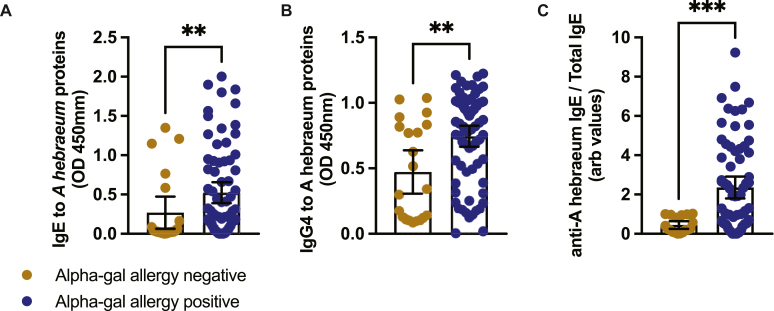
Alpha-gal–allergic patient sera have elevated IgE and IgG4 to *A hebraeum* proteins. **A-C,** Sera from alpha-gal–allergic (n = 72) and nonallergic (n = 21) patients were analyzed for anti–*A hebraeum* IgE (Fig 4, *A*) and IgG4 (Fig 4, *B*) and anti–*A hebraeum* IgE/total IgE ratio (Fig 4, *C*) by ELISA. Experiments were carried out in triplicates (mean ± SEM). Statistical significance was calculated by the Mann-Whitney *U* test. ∗*P* ≤ .05; ∗∗*P* ≤ .01. *OD*, Optical density.

**Fig 5:**
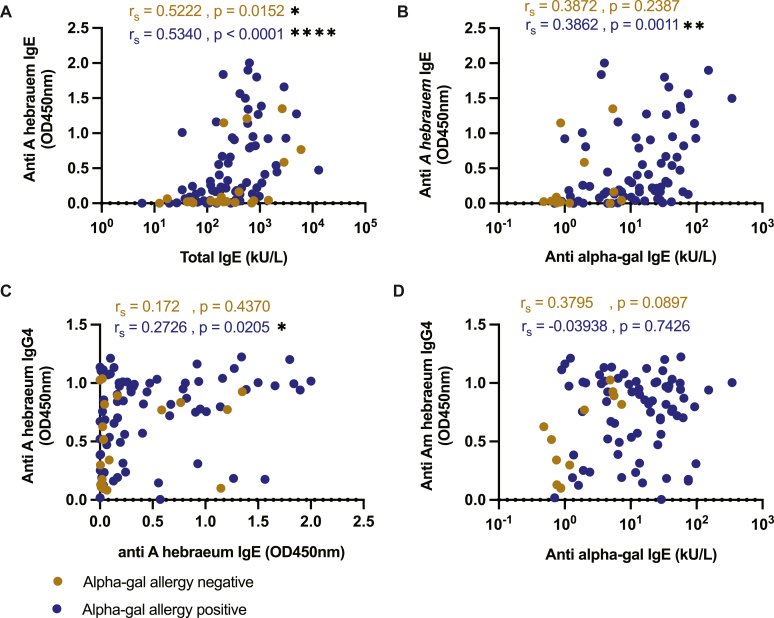
There is a correlation between total IgE, anti–alpha-gal IgE, and anti–*A hebraeum* IgE in sera from alpha-gal–allergic patients. **A-D,** Spearman correlation analysis of anti–*A hebraeum* IgE against total IgE (Fig 5, *A*), anti–*A hebraeum* IgE against anti–alpha-gal IgE (Fig 5, *B*), anti–*A hebraeum* IgG4 against anti–*A hebraeum* IgE (Fig 5, *C*), and anti–*A hebraeum* IgG4 against anti–alpha-gal IgE (Fig 5, *D*) in sera from alpha-gal–allergic (n = 72) and nonallergic (n = 21) patients. Experiments were carried out in triplicates (mean ± SEM). Statistical significance was calculated by the Spearman rank correlation (*r*_s_). ∗*P* ≤ .05; ∗∗*P* ≤ .01. *OD*, Optical density.

Exposure to ticks was assessed by detecting anti–*A hebraeum* IgE and IgG4 in patient serum. Anti–*A hebraeum* IgE and IgG4 were significantly raised in sera from the alpha-gal–allergic group in comparison with the nonallergic group (Fig 4, *A* and *B*). Anti–alpha-gal IgE levels were almost 2-fold higher in the alpha-gal–positive group than in the alpha-gal–negative group (mean, 0.52 vs 0.27; approximately 93% increase; Fig 4, *A*). Similarly, anti–alpha-gal IgG4 levels were elevated in the alpha-gal–positive group (mean, 0.75 vs 0.47) by approximately 60% (Fig 4, *B*). Moreover, anti–*A hebraeum* IgE/total IgE ratio was significantly elevated in sera of alpha-gal–allergic patients in comparison with those of the nonallergic group (mean, 2.36 vs 0.44; approximately 436% increase, ie, 5.4-fold higher) (Fig 4, *C*). There was a moderate-level correlation between anti–*A hebraeum* IgE and total IgE in the allergic group (*r*_s_ = 0.5340; *P* ≤ .0001) and the nonallergic group (*r*_s_ = 0.5222; *P* = .0152) (Fig 5, *A*). A positive correlation between anti–*A hebraeum* IgE and anti–alpha-gal IgE was observed in the alpha-gal–allergic group (*r*_s_ = 0.3862; *P* = .0011) (Fig 5, *B*). Similarly, in sera from alpha-gal−allergic individuals, anti–*A hebraeum* IgE positively correlated with anti–*A hebraeum* IgG4 (*r*_s_ = 0.2726; *P* = .0205) (Fig 5, *C*). In both the allergic and nonallergic groups, the correlation between anti–*A hebraeum* IgG4 and anti–alpha-gal IgE was not significant (Fig 5, *D*).

**Fig 6:**
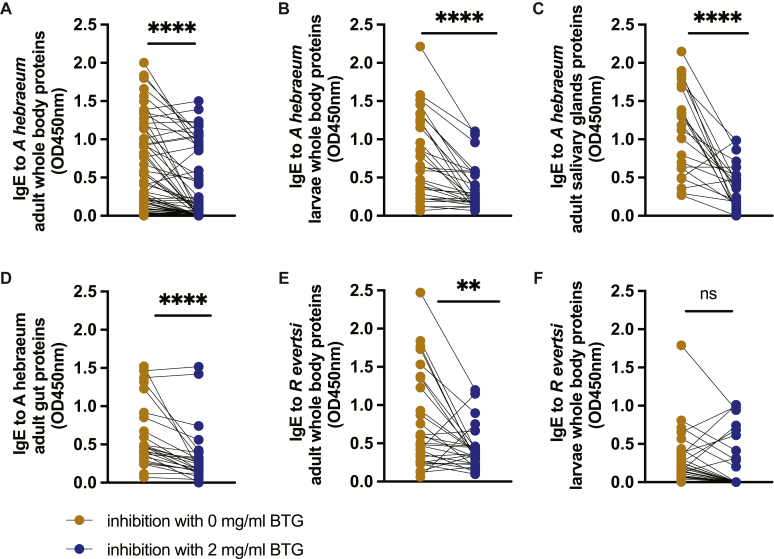
There is reduced binding to proteins from unfed *A hebraeum and R evertsi* after sera anti–alpha-gal IgE competitive inhibition with BTG. **A-F,** Sera (n = 80) were incubated with and without 2 mg/mL of alpha-gal–rich BTG onto ELISA plates coated with 50 μg/mL of *A hebraeum* adult whole-body proteins (Fig 6, *A*), unfed adult salivary gland proteins (Fig 6, *B*), unfed adult gut proteins (Fig 6, *C*), and unfed larval whole-body proteins (Fig 6, *D*) as well as *R evertsi* adult whole-body proteins (Fig 6, *E*) and unfed larval proteins (Fig 6, *F*). Experiments were carried out in triplicates (mean ± SEM). Statistical significance was calculated by the Wilcoxon test. *NS*, Not significant. ∗∗*P* ≤ .01; ∗∗∗∗*P* ≤ .0001. *OD*, Optical density.

To investigate whether sera binding to *A hebraeum* and *R evertsi* proteins is independent of alpha-gal expression on tick proteins, we competitively inhibited sera anti–alpha-gal IgE binding by incubation with BTG, a rich source of alpha-gal, which impairs anti–alpha-gal IgE in patient serum from binding to the alpha-gal antigen. There was a decrease in binding to both *A hebraeum* (adult and larval whole tick lysate, unfed salivary glands, and unfed guts) and *R evertsi* adult whole-body proteins after anti–alpha-gal IgE inhibition. No significant decrease in binding to *R evertsi* larval proteins was observed (Fig 6).

## Discussion

When feeding on a nonprimate mammalian host, tick salivary compounds enable host immune suppression. In humans, exposure to tick alpha-gal glycosylated antigens during feeding is likely to result in alpha-gal sensitization.^18^ We report an increase in the prominence of bands from *A hebraeum* and *R evertsi* salivary proteins binding with anti–alpha-gal antibodies as feeding time increased. Gut proteins also show a similar result with the development of a protein band at 70 to 100 kDa, particularly when the ticks are partially fed. This band disappears in gut proteins from fully fed *R evertsi*. In *A hebraeum* gut proteins, both the bands (130 kDa and 70-100 kDa) disappear when the ticks are fully fed. This demonstrates a system of differential expression of alpha-gal glycosylated proteins during feeding, predominantly with increased alpha-gal glycosylated proteins appearing in salivary glands.^19^^,^^20^ Our data are consistent with studies by Park et al,^21^ which show an increase in the repertoire of salivary gland N-glycans with alpha-gal during feeding.

Similarly, adult and larval stages of *A hebraeum* and *R evertsi* demonstrated differential expression of alpha-gal, with larvae having bands only at 130 kDa. The detection of alpha-gal in unfed larvae may be due to alpha-gal–carrying vitellogenins allowing for transovarian alpha-gal transfer from the adult stage.^22^ As such, alpha-gal–containing proteins in both the larval and adult stages of *A hebraeum* and *R evertsi* suggest ticks in all developmental stages to have the ability to cause alpha-gal sensitization. Our inability to detect alpha-gal in guts and salivary glands from unfed *R evertsi* may be due to the amount of protein loaded being below the level of detection because minimal protein was also observed in these samples by Coomassie staining. However, reevaluation of these samples was not carried out because of limited sample availability.

In line with our previous findings,^14^ here we show immunolocalization of alpha-gal in salivary glands of laboratory-reared *A hebraeum* and *R evertsi* reared to different feeding stages. This is in line with observations from other studies.^8^^,^^22^ Interestingly, unfed male *R evertsi* stained positive for alpha-gal in cell structures reported to contain polysaccharide granules in unfed *R sanguineus* male ticks during the feeding secretory cycle, a phase of physiologic and biochemical changes in the salivary glands before feeding.^23^ Because the ticks used in our study were fed on nonprimate mammalian hosts, it is expected to see staining of the blood meal in the partially fed ticks. However, this was not the case. The red-brownish mass observed is likely to be hematin particles from a digested blood meal. Interestingly, *A hebraeum* gut proteins show no visible alpha-gal bands for alpha-gal in the fully fed state. This may be reflective of the degradation of host-derived alpha-gal–bearing proteins as shown by the ability of ticks to break down host proteins and incorporate them into tick proteins once engorgement is complete.^24^ This can be through the use of heme lipoproteins and/or enzymes such as α-d-galactosidase.^5^^,^^19^^,^^25^

Immunofluorescence experiments by Fischer et al^22^ show colocalization of alpha-gal and clathrin staining, indicating the adsorption of blood meal during hematophagy by endocytosis in clathrin-coated pits. However, the restricted localization of alpha-gal in salivary glands suggests ticks endogenously produce alpha-gal. Consistent with this, some ticks species express alpha-gal transferase genes, namely, *B4GALT7*, *A4GALT-1*, and *A4GALT-2*, and the transcription of these genes is upregulated during feeding.^26^ This upregulation likely increases the levels of corresponding enzymes, resulting in elevated expression of alpha-gal–bearing antigens during blood feeding. Interestingly, unfed male ticks (reared in the laboratory) stained for alpha-gal in their testes, whereas this staining was not present in fed male ticks collected from Mqanduli.^14^ Studies show that *A hebraeum* male ticks attach to a host and feed for about 6 days or until maturity at which point they release pheromones that attract female ticks to attach to the host and start feeding.^27^ *A hebraeum* female ticks do not attach to a host without these signals from the male. Hence, as females feed, mating occurs, and thus fed male ticks release the contents of their testes during the reproductive act. Apart from feeding, this may implicate alpha-gal as essential for mating in ticks.

We report raised IgE to *A hebraeum* and anti–*A hebraeum* IgE/total IgE ratio in sera from the alpha-gal–allergic group. We also demonstrate a moderate and low positive correlation to total IgE and anti–alpha-gal IgE, respectively. Competitive inhibition of anti–alpha-gal IgE reduced binding to unfed *A hebraeum* (adult and larval whole tick lysate, tick salivary glands, and tick guts) and *R evertsi* adult whole-body proteins. This may indicate a link between exposure to *A hebraeum* and/or *R evertsi* tick bites and the development of IgE to alpha-gal and loss of tolerance to dietary alpha-gal resulting in AGS. Choudhary et al^28^ demonstrated the ability of *A americanum* tick salivary gland extract to induce an increase in total IgE at days 0, 7, 21, 28, and 56 after an intradermal injection in mice. They also showed the development of anti–alpha-gal IgE at day 56 in the alpha-gal knockout mice treated with tick salivary gland extract. Similarly, subcutaneous immunization of *A americanum* whole tick extract coupled to an exogenous synthetic alpha-gal glycoprotein has been seen to induce an increase in anti–alpha-gal IgE.^29^ Interestingly, among the nonallergic controls, 12 of 20 participants displayed IgE to alpha-gal with no symptoms after oral food challenge, yet only 3 of these also had detectable IgE to tick proteins (data not shown). This observation suggests that sensitization to alpha-gal alone is not sufficient to drive clinical allergy, and that cosensitization to tick proteins may contribute to the transition from asymptomatic sensitization to symptomatic disease. Hence, alpha-gal allergy may require not only the presence of alpha-gal specific IgE but also additional immune priming through tick-derived antigens.

Anti–*A hebraeum* IgG4 was significantly elevated in the alpha-gal–allergic group as compared with the nonallergic group. Because IgG4 is a marker of chronic exposure, it may suggest high frequencies of tick bites in the alpha-gal–allergic group.^30^^,^^31^ Our cohort is located in a rural environment where there is constant interaction with the environment and domesticated animals. Before attaching onto their host, *A hebraeum* is found close to the soil surface and exhibit active host-seeking behavior when a host is detected (*A hebraeum* does not quest on vegetation; it is an active hunter that hides on the ground and seeks out its host by horizontal locomotion via detection of CO_2_ and body heat). Thus, a rural environment is ideal for increased host-tick interactions. Domestic animals being in close vicinity also make our cohort easily accessible by ticks and hence the raised *A hebraeum* IgG4 in the allergic group. Studies by Hashizume et al^32^ show that patients with 2 or more tick bites have higher levels of anti–alpha-gal IgE, greater basophil and eosinophil infiltration at the site of tick bite, and increased type 2 cytokine T-cell infiltration in comparison with individuals with only 1 or no tick bites. Other researchers show that anti–alpha-gal IgE develops only after successive tick bites and the level of anti–alpha-gal IgE seems to decrease after prolonged periods without tick bites.^33^, ^34^, ^35^ We thus suggest that repeated exposure to *A hebraeum* bites leads to the development of alpha-gal IgE in our alpha-gal–allergic group.

### Conclusions

Several tick species have been identified as clinically relevant for the development of AGS in populations across North and South America, Europe, and Asia; however, there is still no specific tick definitively linked to the onset of clinical AGS in Africa. To rectify this gap, we investigated ticks endemic to a uniquely defined AGS cohort in Mqanduli district, South Africa. Our findings show differential alpha-gal expression in *A hebraeum* and *R evertsi* salivary glands and gut proteins during feeding, with increased binding with anti–alpha-gal antibodies being proportional to feeding time in the salivary glands. Localization of alpha-gal was restricted to salivary acini in both unfed and partially fed *A hebraeum* and *R evertsi.* Sera from alpha-gal–allergic individuals demonstrate raised IgE and IgG4 to *A hebraeum* proteins and a positive correlation between anti–*A hebraeum* IgE and anti–alpha-gal IgE. This indicates a possible role of *A hebraeum* and *R evertsi* tick exposure at any feeding and developmental stage in causing alpha-gal sensitization within our AGS cohort in South Africa.

**Clinical implications**

There is a causal link between repeated bites from endemic ticks and the development of AGS, highlighting tick exposure as a key risk factor in alpha-gal sensitization.

## Disclosure statement

This study was supported by the Birmingham-Nottingham Strategic Collaboration Fund (to W.H. and F.H.F.), the LOEWE Centre DRUID within the Hessian Excellence Initiative (to F.H.F.), a National Research Foundation Innovation M&D Scholarship (to T.M.), and the Allergy Society of South Africa (to W.H).

Disclosure of potential conflict of interest: The authors declare that they have no relevant conflicts of interest.
